# Kuopio birth cohort – design of a Finnish joint research effort for identification of environmental and lifestyle risk factors for the wellbeing of the mother and the newborn child

**DOI:** 10.1186/s12884-018-2013-9

**Published:** 2018-09-21

**Authors:** Pasi Huuskonen, Leea Keski-Nisula, Seppo Heinonen, Sari Voutilainen, Tomi-Pekka Tuomainen, Juha Pekkanen, Jussi Lampi, Soili M Lehto, Hannariikka Haaparanta, Antti-Pekka Elomaa, Raimo Voutilainen, Katri Backman, Hannu Kokki, Kirsti Kumpulainen, Jussi Paananen, Kirsi Vähäkangas, Markku Pasanen

**Affiliations:** 1 School of Pharmacy, Faculty of Health Sciences, University of Eastern Finland, P.O. Box 1627, FI-70211 Kuopio, Finland; 2 Department of Obstetrics and Gynaecology, Kuopio University Hospital, FI-70211 Kuopio, Finland; 3 Obstetrics and Gynaecology, University of Helsinki and Helsinki University Hospital, FI-00029 Helsinki, Finland; 4 Institute of Public Health and Clinical Nutrition, Faculty of Health Sciences, University of Eastern Finland, FI-70211 Kuopio, Finland; 5 Department of Public Health, University of Helsinki, FI-00014 Helsinki, Finland; 6 Department of Health Protection, National Institute for Health and Welfare, FI-70210 Kuopio, Finland; 7 Department of Psychiatry, Institute of Clinical Medicine, University of Eastern Finland and Kuopio University Hospital, FI-70211 Kuopio, Finland; 8 Institute of Behavioural Sciences, University of Helsinki, FI-00014 Helsinki, Finland; 9 Department of Neurosurgery, Institute of Clinical Medicine, University of Eastern Finland and Kuopio University Hospital, FI-70211 Kuopio, Finland; 10 Department of Paediatrics, Kuopio University Hospital, FI-70211 Kuopio, Finland; 11 Department of Anaesthesia and Operative Services, Kuopio University Hospital and Institute of Clinical Medicine, University of Eastern Finland, FI-70211 Kuopio, Finland; 12 Department of Child Psychiatry, Institute of Clinical Medicine, University of Eastern Finland and Kuopio University Hospital, FI-70211 Kuopio, Finland; 13 Department of Biomedicine, University of Eastern Finland and Kuopio University Hospital, FI-70211 Kuopio, Finland

**Keywords:** Birth weight, Environmental health, Foetus, Maternal smoking, Mental health, Metabolism, Nutrition, Paediatrics, Placenta, Research ethics

## Abstract

**Background:**

A Finnish joint research effort Kuopio Birth Cohort (KuBiCo) seeks to evaluate the effects of genetics, epigenetics and different risk factors (medication, nutrition, lifestyle factors and environmental aspects) during pregnancy on the somatic and psychological health status of the mother and the child.

**Methods:**

KuBiCo will ultimately include information on 10,000 mother-child pairs who have given their informed consent to participate in this cohort. Identification of foetal health risk factors that can potentially later manifest as disease requires a repository of relevant biological samples and a flexible open up-to-date data handling system to register, store and analyse biological, clinical and questionnaire-based data. KuBiCo includes coded questionnaire-based maternal background data gathered before, during and after the pregnancy and bio-banking of maternal and foetal samples that will be stored in deep freezers. Data from the questionnaires and biological samples will be collected into one electronic database. KuBiCo consists of several work packages which are complementary to each other: Maternal, foetal and placental metabolism and omics; Paediatrics; Mental wellbeing; Prenatal period and delivery; Analgesics and anaesthetics during peripartum period; Environmental effects; Nutrition; and Research ethics.

**Discussion:**

This report describes the set-up of the KuBiCo and descriptive analysis from 3532 parturients on response frequencies and feedback to KuBiCo questionnaires gathered from June 2012 to April 2016. Additionally, we describe basic demographic data of the participants (*n* = 1172). Based on the comparison of demographic data between official national statistics and our descriptive analysis, KuBiCo represents a cross-section of Finnish pregnant women.

## Background

Population-based pregnancy cohorts have demonstrated that foetal growth, exposure to smoking and pollutants as well as the presence of maternal diseases, the domestic environment, psychological stress and nutrition have long-term effects on the health of the offspring [1, 2]. One way to investigate the multiple associations between the effects of prenatal exposure and health of the offspring later in life is via birth cohorts that combine clinical, questionnaire and biological data in a way that is easy to link. According to the Declaration of Helsinki (www.wma.net) and legislation in many countries including Finland (488/1999; www.finlex.fi), research ethics in human studies requires special attention. However, how to carry out practical procedures in cohort studies in ethically best way is not self-evident and requires further studies [3].

Human foetus is much more sensitive than adult to the adverse effects of chemicals or other risk factors. This is due to the on-going development, characterized by high cell proliferation capacity combined with deficiencies in the foetal metabolic detoxification capacity, an immature immune system and still-developing DNA-repair mechanisms [4]. At this stage, chemicals can permanently reprogram physiological responses through epigenetic mechanisms increasing the susceptibility of the foetus to diseases appearing after birth [5]. These modifications can be analysed, for instance, by RNA sequencing, metabolomics, proteomics and epigenetics [6–8]. In addition, it has also been demonstrated that maternal health status during pregnancy predicts future wellbeing of the child and mother [9].

Alterations occurring during the pre-, peri- and postnatal developmental periods can increase sensitivity for further aberrations at later stages of life [10]. Human data supporting this hypothesis emerges from studies showing that aberrant programming of growth and development can lead to respiratory disease [11], non-Hodgkin lymphoma [12] and neurological diseases [13].

In Finland, approximately 50% of pregnant women consume medicinal products during their pregnancy [14]. In addition to medicinal products, natural and herbal medications are frequently used during pregnancy, and should be considered as a possible foetal risk factor [15]. Furthermore, up to 15% of pregnant women smoke in Finland [16]. There is a paucity of data concerning various maternal exposures during pregnancy and their impact on overall developmental outcomes.

Nutrients and their metabolism play a crucial role in the health and wellbeing of both mother and foetus, and they are associated with the long-term health of the offspring [17]. Less information exists about the intake of non-essential nutrients and other food-based compounds and about their association with the health of children.

Kuopio Birth Cohort (KuBiCo, www.kubico.fi) is anchored on the concept of developmental origin of diseases [18–20]. It will generate new knowledge by integrating clinical and analytical data, achieving a systematic increase in understanding the combined effects of multiple factors on health and disease. KuBiCo is intended to help resolve the effects of genetic and epigenetic backgrounds and potential risk factors (medications and nutritional, lifestyle, and environmental factors) during pregnancy on the health status of the mother and child. KuBiCo is a joint research effort between the University of Eastern Finland (UEF), the Kuopio University Hospital (KUH) and the National Institute for Health and Welfare (THL).

This paper describes the setup of KuBiCo and descriptive analysis of response frequencies and feedback to KuBiCo questionnaires of altogether 3532 parturients from June 2012 to April 2016. Additionally, we report basic demographic data of 1172 KuBiCo participants and show that they agree with Finnish national statistics.

## Methods/design

Altogether, the final database is planned to include 10,000 mother-child pairs. All pregnant women who are expected to give birth in KUH in the Finnish county Northern Savo are invited to participate in KuBiCo. KuBiCo does not include individual exclusion criteria. Those who sign the informed consent at any stage of pregnancy will be included in KuBiCo and prospective data collection. Majority of the participants (> 90%) are recruited during the routine first trimester visit (gestational week 6–9) at the prenatal clinics by primary health care personnel or midwifes, when the pregnancy has been determined by clinical inspection.

### Collection and organization of data and samples

**Fig. 1:**
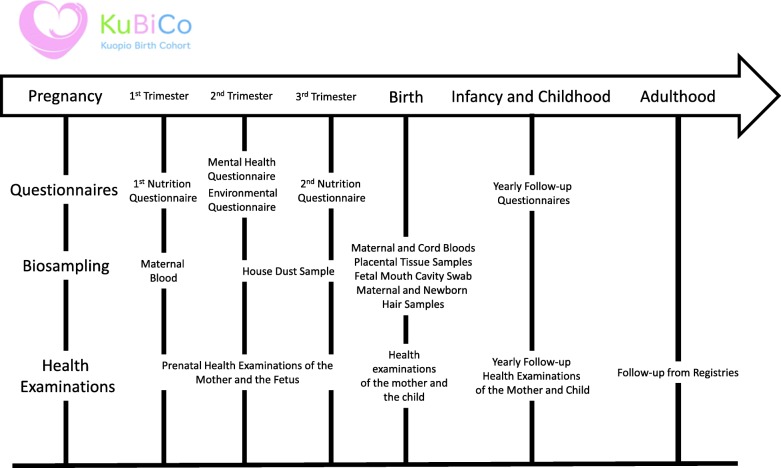
Data collections and timeline of KuBiCo

**Fig. 2:**
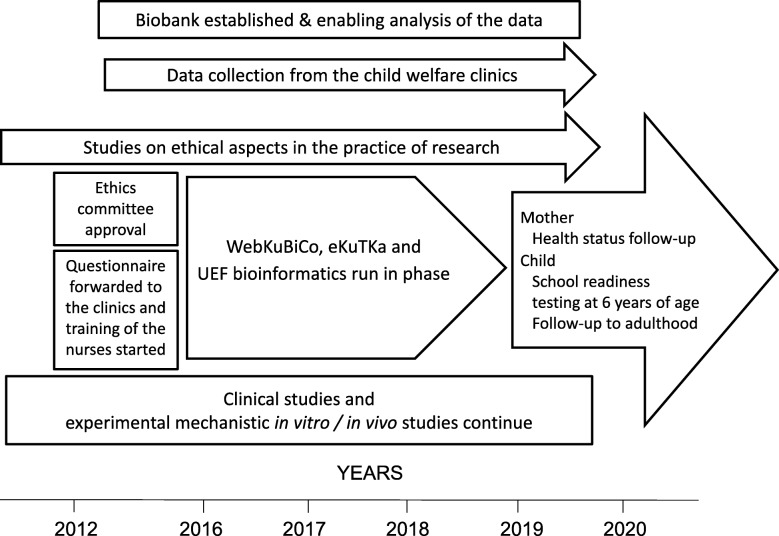
Outline of the KuBiCo. Questionnaire and information platform (WebKuBiCo), electronic KuBiCo database (eKuTKa), University of Eastern Finland (UEF)

Barcode identification stickers with a KuBiCo code are used to process samples into the system and archive all biological samples (maternal and umbilical cord blood, placental samples, microbial swab from neonate’s oral cavity, hair and house dust samples) (Fig. 1). The groups participating in KuBiCo will utilize the WebKuBiCo database that sends, receives and stores all the questionnaire data from the beginning of the confirmed pregnancy until the child reaches adulthood. Each woman is entered into the WebKuBiCo database by using her personal bank account safety codes (the most widely used electronic identification system in Finland) and each pregnancy will have a KuBiCo code of its own. Therefore, during the project, one woman may have several KuBiCo codes in relation to subsequent pregnancies. This web-based platform will also automatically create educative/guiding feedback to the women based on their answers to the questionnaires. So far, the service has been developed for mental health and nutrition questionnaires. An outline of the KuBiCo is presented in Fig. 2.

Biological samples (maternal and umbilical cord blood, placental tissue sample, microbial swab from neonate’s oral cavity, maternal and foetal hair samples) are collected by staff in KUH and the main management of samples is conducted by personnel of UEF. House dust samples are collected by the participating families according to THL instructions. Biological samples are stored in deep-freezers (− 80 °C) of KUH, UEF, and THL. House dust samples are stored and managed in the THL facilities.

All raw data from the questionnaires and biological samples will be collected and stored in an electronic KuBiCo database (eKuTKa) from which data can be downloaded for investigation by all research partners and international collaborators after mutual agreements. The KuBiCo advisory board grants permissions of KuBiCo data usage for research groups. This database will be developed and maintained by ISTEKKI (Information Technology services of KUH). The Bioinformatics unit at the UEF provides the appropriate bioinformatics software licenses, such as Revolution R Enterprise and Ingenuity Pathway Analysis (IPA). All raw data obtained from our own or any collaborative studies will be stored on the eKuTKa database and analysed by the core facilities. This will ensure continuous support for high quality research.

Data collected in the work packages is stored in centralized data servers. These consist of raw data, pre-processed data, and selected results from data analysis. Central storage and processing enables efficient curation, harmonization and integration of the data, resulting in high quality databank that can be reliably used for a wide range of research purposes. The data is version controlled and backed up, ensuring efficient storage and re-use. Access to specific parts of the data will be controlled, allowing for functional and secure data sharing and dissemination between the research partners and the research community as a whole. Standard formats for handling data from different high-throughput technologies will be used, and such data will be submitted to open access databases, such as Gene Expression Omnibus (GEO), as required by scientific journals upon publication.

To ensure harmonization of the research protocols for the studies performed in the different work packages of the KuBiCo, all active partners have approved final protocols. This will lead to standardized collection, handling and storage procedures of the biological samples by the research groups. This also applies to the questionnaires on intake of food and supplements, somatic and mental health, ethics and all developmental follow-ups. In order to facilitate planned future multinational collaborative research, all questionnaires are being translated into English from the original Finnish versions. By standardizing all the protocols and questionnaires, optimally robust and sufficiently powered statistical analyses will be achieved. Power calculations for the KuBiCo are conducted separately within each individual work package or research hypothesis using KuBiCo data. In addition, any missing data is handled separately in each KuBiCo work package. During study and after completion of the material collection the KuBiCo database can be linked to national health registries for epidemiological follow-ups.

### Research ethics

The Research Ethics Committee of Hospital District of Central Finland in Jyväskylä, Finland has reviewed and approved the KuBiCo plan 15.11.2011. The ethical basis of KuBiCo is the expected major benefit for future research projects on developmental origin of various childhood and adult-onset diseases and the health of future generations. Data sharing among the participating research groups will make it possible to undertake large projects, and furthermore, all of the developed data will remain in the database for future generations of scientists. Pregnant women are asked to consider volunteer participation, which will involve that they provide their data and samples. In return, they are automatically informed about their nutritional or mental health status if any causes of concern are identified.

### Work packages within KuBiCo

*Metabolism and omics* will study how different risk factors affect placental hormone production (i.e., potential for endocrine disruption) and xenobiotics metabolizing characteristics at different stages of pregnancy by the means of metabolic activity determination, genetics and epigenetics.

*Paediatrics* will survey the somatic health, growth and overall development of the children by annual questionnaires to elucidate the role of prenatal factors, for example maternal smoking and microbe exposure during birth, on health and morbidity of the offspring in later childhood, adolescence and adulthood.

*Mental wellbeing* targets associations between maternal psychological wellbeing and systemic biomarkers during pregnancy, and how the maternal mental wellbeing modulates neonate physiology and the child’s later development. Furthermore, the psychological development and wellbeing of the child as well as parent-child relationships will be studied.

*Prenatal period and delivery* evaluate perinatal and genetic epidemiology, and risk factors and markers for most common obstetrical and perinatal complications, and how these factors affect the perinatal outcomes and later the child’s subsequent health. Data is collected on different prenatal exposures by biological samples and questionnaires and linked to the information available on prenatal outcomes and the child’s subsequent health.

*Analgesics and anaesthetics during peripartum period* evaluates the foetal and newborn effects of analgesics and anaesthetics given for parturient during the perinatal period. Maternal pharmacokinetics and –dynamics of different compounds used for pain relief and anaesthesia will be measured aimed at establishing a pharmacodynamic and -kinetic in silico model based on foetal and newborn exposures evaluated from venous blood.

*Environment* The studies on the effects of different environmental exposures are focused on environmental determinants of asthma and allergic diseases, especially of the exposure to the microbes in indoor air and air pollution, and of the environmental chemical exposure on the health and development of the offspring. Environmental exposures are assessed in KuBiCo with two separate comprehensive questionnaires during first and third trimesters. In addition, voluntary house dust samples are collected during first and third trimesters and neonatal mouth mucosa swab at birth. Also, maternal serum samples are collected for chemical analyses during first trimester and at birth, and umbilical cord blood samples at birth if clinically feasible (see Table 1).

**Table 1:** Number of participants answering KuBiCo questionnaires from June 2012 to April 2016

General data	Total number of participants (per cent of total number of parturients at Kuopio University Hospital)	3532 (37.3%)
	Number of children included in the follow-up study	3187
	Women participating in KuBiCo for the second time	230
Number of women answering questionnaires of	Ethics	2635
	Environmental stress factors – first trimester/last trimester	2464/2134
	Nutrition up to gestation week 13/from gestation week 28 to delivery	1625/3240
	Mental health	2658
	When the child was 12 months old	1955
	When the child was 24 months old	1477
	Nutrition when the child was 24 months old	1402
	When the child was 36 months old	480
Number of women collecting samples	Women who completed the collection of house dust	630

*Nutrition* Intake of food and supplements are collected using food frequency questionnaire (FFQ) during the first and third trimester of pregnancy and dietary data of the children at 2 and 4 years of age. Web-based FFQ comprises a food list of about 160 food items with nine frequency response options from “never” to “six or more times per day”. More than 60 nutrients and 100 food groups are provided. FFQ includes also specific questions about supplement use. KuBiCo FFQ has been further developed to produce personal feedback about nutrition at first and third trimester of pregnancy.

*Research ethics* KuBiCo provides a unique opportunity to pursue the following important points: 1) recruitment and informed consent in a birth cohort, 2) evaluation by the recruited families of the usefulness and potential risks to their families, 3) handling and long-term storage of samples and information for future use, 4) societal benefits of the type of information collected in KuBiCo and 5) what affects the decisions of the people approached for recruitment. One of the main aims is to develop reliable tools to evaluate ethical issues in birth cohort studies.

### Descriptive analysis of the KuBiCo database

All material collected between June 2012 and April 2016, altogether 3532 maternal answers to the questionnaires were included in the descriptive analysis. Additionally, an analysis of demographic data of mother-child pairs was carried out. This included full background information and medical records containing 1172 pregnancies, which included 1156 single pregnancies and 16 twin pregnancies. The background information collected from the mothers included age, number of medications, diseases, smoking status, and use of alcohol during pregnancy. Information about pregnancies included the duration of the pregnancy and the weight of placenta. The newborn information included birthweight, gender and date of birth. The overall participation in KuBiCo during 2012–2016 (3532 women) has been 37.3% of all parturients giving birth in KUH. Table 1 describes the number of women who filled KuBiCo forms completely or partly and Table 2 the descriptive characteristics of the cohort.

**Table 2:** Characteristics of the study population in relation to the gestational age at delivery

	Total newborn *n* = 1172	Preterm birth < 37 GWS *n* = 81 (6.9)	Term birth 37–40 GWS *n* = 855 (73.0)	Late-term birth ≥41 GWS *n* = 236 (20.1)
Birth weight, g	3446 ± 597	2156 ± 704	3497 ± 470	3701 ± 395
1st tertile ≤3280		79 (20)	218 (56)	94 (24)
2nd tertile 3281-3700		2 (0.5)	166 (43)	223 (57)
3rd tertile ≥3701		0 (0)	131 (34)	259 (66)
Placental weight^a^, g	590 ± 130	439 ± 117	592 ± 125	620 ± 124
1st tertile ≤530		51 (13)	179 (47)	155 (40)
2nd tertile 531–630		8 (2.1)	179 (47)	196 (51)
3rd tertile ≥631		2 (0.5)	145 (39)	225 (61)
Maternal age^b^, years	29.8 ± 5.1	30.7 ± 5.4	29.9 ± 5.0	29.4 ± 5.3
1st tertile ≤28		21 (4.6)	203 (44)	233 (51)
2nd tertile 29–32		28 (7.4)	161 (43)	188 (50)
3rd tertile ≥33		22 (6.8)	145 (45)	155 (48)
Number of previous deliveries^b^	1.0 ± 1.3	0.7 ± 1.1	1.1 ± 1.3	0.7 ± 1.1
Nulliparous		45 (8.8)	210 (41)	258 (50)
Primiparous		14 (3.9)	159 (44)	186 (52)
Multiparous		12 (4.2)	140 (49)	132 (47)
Number of twin pregnancies	16 (1.4)	10 (63)	6 (38)	0 (0)
Gestational week at delivery, weeks^b^	39.2 ± 2.0	33.5 ± 3.2	39.1 ± 0.9	41.1 ± 0.3
Foetal sex, males	589 (50.3)	42 (51.9)	443 (51.8)	104 (44.1)
Self-reported smoking before pregnancy	226 (20)			
Number of daily cigarettes before pregnancy^b^				
No smoking	957 (80)	60 (6.3)	715 (75)	182 (19)
1–10	135 (11.7)	4 (3.0)	93 (69)	38 (28)
≥ 11	64 (5.5)	7 (11)	41 (64)	16 (25)
Self-reported smoking during pregnancy	49 (4.2)			
Number of daily cigarettes during pregnancy^b^				
No smoking	1104 (96)	68 (6.2)	811 (74)	225 (20)
1–5	28 (2.4)	1 (3.6)	22 (79)	5 (18)
≥ 6	24 (2.1)	2 (8.3)	16 (67)	6 (25)
Self-reported alcohol consumption before pregnancy^b^, weekly doses (average)				
None	601 (52)	27 (4.5)	468 (78)	106 (18)
1–5	497 (43)	40 (8.0)	343 (69)	114 (23)
≥ 6	58 (5.0)	4 (6.9)	38 (66)	16 (28)
Self-reported alcohol consumption during pregnancy^b^, monthly (average)				
Never	1053 (96)	66 (6.3)	172 (73)	215 (20)
Less than once	36 (3.3)	12 (2.8)	23 (64)	12 (33)
Once a month, and more	11 (1.0)	1 (9.1)	9 (82)	1 (9.1)

Values are n (%) or mean ± SD

^a^Excluded twin pregnancies, *n* = 16

^b^mothers with twin pregnancies included only once, total *n* = 1156 gestational weeks (GWS)

Approximately one-fifth of the women had taken at least one regular medication during pregnancy (Table 3). Regular medications were mainly related to the women’s chronic diseases; thyroid diseases, asthma and mental health conditions which were the most frequent chronic diseases in pregnant women as expected (Table 4).

**Table 3:** Regular use of medication among 1156 women during pregnancy in relation to gestational age at delivery

Amount of regular medication	Total *n* = 1156	Preterm birth < 37 GWS *n* = 71 (6.1)	Term birth 37–40 GWS *n* = 849 (73.4)	Late-term birth ≥41 GWS *n* = 236 (20.4)
No regular medication	928 (80)	52 (6)	683 (74)	193 (21)
Regular medication	228 (20)	19 (8)	166 (73)	43 (19)
1 medication	124 (11)	9 (7)	89 (72)	26 (21)
2 medications	60 (5)	6 (10)	89 (72)	26 (18)
≥ 3 medications	44 (4)	4 (9)	34 (77)	6 (14)

Values are *n* (%) of all parturients. Gestational weeks (GWS)

**Table 4:** Women with specific chronic diseases with regular prescription medications initiated before pregnancy in relation to the gestational age at delivery

	Total *n* = 1156	Preterm birth < 37 GWS *n* = 71 (6.1)	Term birth 37–40 GWS *n* = 849 (73.4)	Late-term birth ≥41 GWS *n* = 236 (20.4)
No regular medication	928 (80)	52 (6)	683 (74)	193 (21)
Thyroid diseases	96 (8)	7 (7)	71 (74)	18 (19)
Asthma	60 (5)	6 (10)	43 (72)	11 (18)
Mental health conditions	45 (4)	4 (9)	32 (71)	9 (20)
Neurological diseases	7 (0.6)	1 (14)	2 (29)	4 (58)
Diabetes	10 (0.9)	3 (30)	7 (70)	0
Inflammatory bowel diseases	13 (1)	0	10 (77)	3 (23)
Hypertension	13 (1)	5 (39)	8 (62)	0

Values are *n* (%) of all parturients. Gestational weeks (GWS)

## Discussion

The organization of the KuBiCo is unique by enabling linkage of birth cohort biobank with clinical follow-up and thus creating the possibility to tackle various questions within the developmental origin of disease concept.

One of the advantages of the KuBiCo is that the Finnish primary health care system routinely monitors the pregnant women and children and records their medical history. The other advantage of KuBiCo is that all of the questionnaires are in an electronic format, and can be filled in whenever convenient for the participant. Every pregnant woman will receive her personal WebKuBiCo password that allows her to fill in the form and to follow the progress of the project. Additionally, some of the questionnaires (mental health and nutrition) include automatic feedback to the participant about their present health status highlighting “safety phrases” if certain threshold scores are exceeded. These thresholds are based on established clinical cut-offs [21] and nutritional guidelines [17]. The aim of this feedback was to motivate and provide an additional health benefit for the participating pregnant women, and they have reported that the feedback has been helpful. Therefore, the developed feedback service is already included into the daily routines in the clinics.

To identify selection bias, a descriptive analysis was performed for 1172 participants of KuBiCo. We found out that KuBiCo corresponds well with the general pattern of Finnish pregnancies and deliveries. In the descriptive analysis (2012–2014), 6.9% of the deliveries were preterm (< 37 weeks), 73% full-term (37–40 weeks) and 20.1% late-term (≥41 weeks). In 2015, the corresponding nationwide Finnish delivery statistics were 5.9% preterm, 71.4% full-term and 22.5% late-term deliveries [16]. Mean maternal age in KuBiCo was 29.8 ± 5.1 years and the Finnish average 30.6 years in 2015. The Finnish mean maternal age has increased by 1.5 years from the 1990s, including older primiparas and increased numbers of over 35-year old women [16].

The mean birth weight in this descriptive analysis was 3446 ± 597 g (*n* = 1172) which is similar to the Finnish mean birthweight i.e. 3485 g in 2015 [16]. Compared to other Scandinavian countries, the decrease of mean birthweight has been more significant in Finland since the 1990s. This decrease of mean birthweight is especially apparent in boys and in macrosomic foetuses. The reason for this phenomenon is unknown and a potential subject for further investigations within and between cohorts.

There were 16 twin pregnancies in this descriptive analysis (1.4% of 1156 deliveries) which is equivalent to the Finnish overall rate of multiple births (13.6/1000 deliveries in 2014) [22]. The twinning rate in Finland is low when compared to European median twinning rate, which was 16.8% in 2010 [23]. Twin pregnancies carry higher risks of adverse foetal and neonatal outcomes such as higher rates of preterm birth, perinatal mortality and in the longer-term neuro-developmental impairments [23]. In this analysis, ten out of the sixteen twins (62.5%) were preterm. One reason for the lower rate of twin pregnancies in Finland may be due to the recommendations of one-embryo transfers in infertility treatment programs.

The gender percentages of the newborns in KuBiCo were 49.7% girls and 50.3% boys (n = 1172). Based on the official statistics in Finland, there have been more boys than girls since 1761 at least until 2015. In the past twenty years, the proportion of boys has varied between 50.9–51.4% in Finland. In 2015, a total of 55,759 children were born in Finland, and there were 1.3% more boys than girls [16]. However, sex ratio changes have been reported from Spain [24], Scotland [25] and USA [26]. In this descriptive analysis, in the group of late-term births, the percentage of boys (44.1%) was lower compared to girls. This signal will need to be confirmed in the future analyses.

During 1996–2010 in Finland, it was reported that nearly every second pregnant woman (46.9%) purchased at least one prescription medication during any trimester of her pregnancy [14]. In our questionnaire-based data, 20% of women had used at least one regular prescription medication during pregnancy. It has been shown that some medications, such as anti-epileptics and antidepressants, may increase the risk for preterm birth and lower birthweight [27]. Lahesmaa-Korpinen and colleagues [14] observed that perinatal risks were more frequent when pregnant women are exposed to any pharmaceuticals during pregnancy. In the same study, the pregnant women who used medications had a 13% higher risk to have a small for gestational age newborn, a 27% higher risk to have a large for gestational age newborn and a 20% higher risk for preterm delivery in comparison with those women with no medications. In accordance with this proposal, we noted that preterm delivery was more frequent among the women with regular medication. However, it should be also stated that most of the women with regular medication delivered at term or post-term.

Cigarette smoking and use of alcohol are obviously the most frequent underestimated, but well recognized, confounding health factors. Fortunately, smoking during pregnancy in Finland is decreasing. In 2012, 17% of pregnant women smoked during pregnancy, but the percentage is decreasing [22]. In KuBiCo, only about 4% of the pregnant women reported smoking during pregnancy. However, when their smoking status was checked by serum cotinine determination in a subset, 8.5% had cotinine in amounts indicating smoking that is comparable to the levels reported previously [22]. Obviously, the present numbers are affected by the selection bias created by e.g. motivation and nicotine replacement therapy. In KuBiCo placental xenobiotic and steroid metabolizing enzymatic analysis in vitro [6, 7], confirms the induction of cytochrome P450 1A1 (CYP1A1) by smoking.

Based on the comparison of demographic data between official national statistics and our descriptive analysis, KuBiCo cohort represents a cross-section of Finnish pregnant women. KuBiCo is a multidisciplinary collaborative effort that enables to unravel the effects of genetics, epigenetics and complex life style factors on the future health status of both mother and child. The KuBiCo is equipped with a digital database (eKuTKa) to serve all contributors; pregnant women can sign in, they can follow the progress and receive feedback from their questionnaires; it also permits the registered investigators’ access the generated data. In its final form, the database will include the data from 10,000 mother-child pairs. Our descriptive analysis demonstrates that the material and the proposed confirmatory measures, for example smoking status verification, will guarantee the reliability and high quality of this cohort so that it can be exploited in future projects and analyses.

## Abbreviations

eKuTKa: electronic Kuopio Birth Cohort database
FFQ: Food frequency questionnaire
GEO: Gene Expression Omnibus
GWS: Gestational weeks
IPA: Ingenuity Pathway Analysis
ISTEKKI: Information Technology services of KUH
KuBiCo: Kuopio Birth Cohort
KUH: Kuopio University Hospital
THL: National Institute for Health and Welfare
UEF: University of Eastern Finland
WebKuBiCo: Questionnaire and information platform
